# No Own-Age Bias in Children’s Gaze-Cueing Effects

**DOI:** 10.3389/fpsyg.2018.02484

**Published:** 2018-12-20

**Authors:** Rianne van Rooijen, Caroline Junge, Chantal Kemner

**Affiliations:** ^1^ Experimental Psychology, Helmholtz Institute, Utrecht University, Utrecht, Netherlands; ^2^Developmental Psychology, Utrecht University, Utrecht, Netherlands; ^3^Brain Center Rudolf Magnus, University Medical Center Utrecht, Utrecht, Netherlands

**Keywords:** gaze cueing, own-age bias, children, attention, eye-tracking

## Abstract

Sensitivity to another person’s eye gaze is vital for social and language development. In this eye-tracking study, a group of 74 children (6–14 years old) performed a gaze-cueing experiment in which another person’s shift in eye gaze potentially cued the location of a peripheral target. The aim of the present study is to investigate whether children’s gaze-cueing effects are modulated by the other person’s age. In half of the trials, the gaze cue was given by adult models, in the other half of the trials by child models. Regardless of the models’ ages, children displayed an overall gaze-cueing effect. However, results showed no indication of an own-age bias in the performance on the gaze-cueing task; the gaze-cueing effect is similar for both child and adult face cues. These results did not change when we looked at the performance of a subsample of participants (*n* = 23) who closely matched the age of the child models. Our results do not allow us to disentangle the possibility that children are insensitive to a model’s age or whether they consider models of either age as equally informative. Future research should aim at trying to disentangle these two possibilities.

## Introduction

The direction of an eye gaze of another person can be very informative. Following the gaze of someone else may lead to detection of important environmental stimuli and the initiation of joint attention (for a review, see Frischen et al., 2007). People’s ability to shift attention in response to another’s eye gaze is often examined with the gaze-cueing paradigm. The classic gaze-cueing experiment shows a face, which makes an eye movement toward either right or left, after which a target appears on one side of the face. People are faster in detecting the target when the eye movement correctly predicted the target location, which is called the gaze-cueing effect (Friesen and Kingstone, 1998). In the current study, we set out to examine whether children’s gaze-cueing effect is affected by whether the other person matches their own age or not.

Gaze cueing appears to be a crucial ability in several learning processes. For instance, shifting attention in response to gaze cues during infancy correlates with subsequent language development (Morales et al., 1998, 2000; Brooks and Meltzoff, 2005). Moreover, faster gaze switching in response to gaze cues is related to less severe social and language symptoms in children with autism (Charman, 2003) and to less severe autistic traits in adults (e.g., Bayliss and Tipper, 2005). Nevertheless, gaze-cueing effects are not always apparent, at least in infancy, as a comparison across gaze-cueing studies with infant samples shows (Munsters, 2017). Studies with older typically developing children show a more robust gaze-cueing effect (Swettenham et al., 2003; Kylliäinen and Hietanen, 2004; Senju et al., 2004; Goldberg et al., 2008; van Rooijen et al., 2018). However, when testing children with autism, the results are again mixed (Swettenham et al., 2003; Kylliäinen and Hietanen, 2004; Senju et al., 2004; Goldberg et al., 2008). These discrepancies could be related to differences in task settings. This suggests that experimental factors, such as stimulus material and gaze cue presentation time or the inter-stimulus interval between gaze cue onset and target onset, contribute to the magnitude of this effect. The current study specifically aims to evaluate whether stimulus characteristics can modulate gaze-cueing effects.

One possible factor of influence on the gaze-cueing effect is the characteristics of the faces which are used as stimuli, such as species, race, age, and gender (cf. Macchi Cassia, 2011). For example, using familiar faces as stimulus models enhances the gaze-cueing effect; yet, this effect is only observed in women (Deaner et al., 2007). Moreover, young adults show larger gaze-cueing effects for young adult stimulus models than older adult stimulus models (Slessor et al., 2010). Although in adults both familiarity and age modulate gaze cueing, it is unclear how these face categories influence sensitivity to gaze cues across development. In particular, when assessing developmental trajectories in gaze cueing from childhood to adulthood (e.g., van Rooijen et al., 2018), it is vital to understand how stimulus characteristics can affect gaze-cueing effects.

The current study will therefore examine whether the age of the perceived stimulus model modulates gaze cueing in a child sample, similar to the effect observed in adults (Slessor et al., 2010). Research reveals that humans easily estimate a person’s age (Rhodes, 2009), which in turn influences the process of face encoding (e.g., Kuefner et al., 2008; Ebner et al., 2010). When adults see other adult faces from various angles, Kuefner et al. (2008) observed a large inversion effect (i.e., recognition of inverted faces is less accurate than recognition of upright faces), whereas this effect is smaller for seeing child faces and even absent for newborn faces. This finding suggests that adults have difficulties in encoding configural information in other age faces, and thus, perceptual strategies are more finely tuned to adult faces. This is often referred to as the own-age bias.

Evidence for an own-age bias in face processing mainly comes from the field of face recognition (for reviews, see Macchi Cassia, 2011; Rhodes and Anastasi, 2012; Wiese et al., 2013). Next to research with the inverted-face paradigm, other studies test face recognition with a learning and a consecutive test phase. In this test phase, the faces of the learning phase are presented, as well as new faces. Per face, the participants make an old/new judgment. Meta-analyses reveal that the own-age bias is a robust effect in this field of research, with higher recognition accuracy for own-age faces compared to other-age faces (Rhodes and Anastasi, 2012). The own-age bias is not only present in adults (e.g., Kuefner et al., 2008) but also in developing populations. Indeed, children show superior recognition of child faces compared to adult faces (e.g., Anastasi and Rhodes, 2005; Lindholm, 2005; Crookes and McKone, 2009) and also adolescents show own-age effects (Picci and Scherf, 2016). One of the explanations for the existence of an own-age bias is that people have more extensive experience with individuals of their own age, and therefore, perceptual processes specifically support own-age faces (Harrison and Hole, 2009; He et al., 2011).

This own-age bias in face recognition appears to be very narrow. When faces of 8-year-old children are used as stimuli, only 7- to 9-year-old children show enhanced recognition for these faces. In contrast, 6- and 11-year-old children are less accurate in recognizing these 8-year-old stimulus faces (Hills and Lewis, 2011). A follow-up study showed that the optimal age range in which an own-age bias in face recognition can be observed might be even smaller. Children were followed from the age of 7 years until they were 9 years old. Each year, children performed a face recognition task in which faces of 8-year-olds were used as stimuli. Children showed better face recognition scores for this set of faces when they were 8-year-olds themselves, compared to their performance when they were 7 or 9 years old (Hills, 2012). Thus, in children, the own-age bias in face recognition seems to be present but highly restrictive.

Although the own-age bias is often present in face recognition studies, there are only a few studies that investigated whether own-age biases are present in the gaze-cueing paradigm. To our knowledge, only two previous studies looked at an own-age bias in the gaze-cueing paradigm, yet both with an adult population. Slessor et al. (2010) investigated whether younger (mean age 20 years) and older (mean age 73 years) adults differed in their gaze-cueing effect while using younger and older adult stimulus models. There was an overall age effect, which showed that irrespective of the age of the stimulus face, older adults show a smaller gaze-cueing effect than younger adults. In addition, the gaze-cueing effect was influenced by an interaction with the age of the stimulus model. The younger adults express an own-age bias, whereas the older adults do not show such a bias (see also Ciardo et al., 2014). Thus, the age difference observed in this study is attributable to the fact that young adults have larger gaze-cueing effects in response to young adult faces. Nonetheless, Bailey et al. (2014) demonstrated a different pattern of own-age biases: when seeing subliminal stimuli or happy facial expressions, only older adults (but not younger adults) exhibit an own-age bias. These studies indicate that an own-age bias can be observed in gaze-cueing paradigms with adults of varying ages, yet stimulus presentation time and the addition of emotional expressions to the stimulus set seem to alter the results. As the current study is the first to examine own-age effects in gaze cueing in a child sample, we will apply the more basic design and use supraliminal stimuli with neutral expressions. In this way, we are also able to directly compare our results with the adult data of Slessor et al. (2010).

Surprisingly, it appears that gaze-cueing studies with children employ only adult faces (e.g., Swettenham et al., 2003; Kylliäinen and Hietanen, 2004; Senju et al., 2004; Freeth et al., 2010a,b; Riby et al., 2013; van Rooijen et al., 2018). However, children might simply be more tuned toward processing faces of their own age group, as is suggested by the enhanced face recognition of own-age faces (Anastasi and Rhodes, 2005; Lindholm, 2005; Crookes and McKone, 2009; Hills and Lewis, 2011; Hills, 2012; Picci and Scherf, 2016). This enhanced processing of own-age faces might in turn boost their performance on a gaze-cueing task that uses child models as stimuli. If children indeed express an own-age bias in gaze cueing, the use of only adult stimulus models might lead to underestimation of their performance in relation to subjects from other ages, or it might mask true developmental effects in longitudinal studies. Including both child and adult stimuli in a gaze-cueing task thus gives a better representation of a person’s ability to orient one’s own attention in response to gaze cues from a variety of models. The present study therefore explores whether children display an own-age effect in a gaze-cueing paradigm by presenting models who are either adults or children around the age of 10 years.

The present eye-tracking study first investigates whether there is an overall own-age bias in a gaze-cueing paradigm in children from a wide age range (6- to 14-year-olds). As in the study of Slessor et al. (2010), our subjects see supraliminal faces with neutral expressions whose shift in eye gaze either validly or invalidly cues the presence of a peripheral target. Given the literature on the own-age bias in face recognition processes, we hypothesize that children will show a stronger gaze-cueing effect for child models compared to adult models. Another possibility is that own-age effects are present but rather narrow in scope, that is, only present for those children that match the child models closely in age, as is also sometimes observed in face recognition paradigms (i.e., Hills and Lewis, 2011; Hills, 2012). In a secondary set of analyses, we therefore repeat our analyses, focusing solely on children aged between 10 and 11 years.

If we find evidence that children take the age of the person they look at into account when directing their attention, an own-age effect in gaze cueing would manifest itself as larger gaze-cueing effects for child models: children consider gaze cues of other children more informative. Yet another possibility is that we find a larger gaze-cueing effect for the adult models instead. This finding could indicate that children see adult cues as more informative, which results in faster processing and redirection of attention for this category of faces. Alternatively, if we fail to find any effect of the model’s age on children’s responses, there are at least two possibilities that could explain this: either children are insensitive to the model’s age or they find adult and child models equally informative.

## Materials and Methods

### Subjects

A total of 74 children participated in this study (40 boys; *M* age = 10.41 years, *SD* = 1.91, range 6.2–14.4 years). Another two children were tested but were excluded because of difficulties with the eye tracker to detect the pupil. We created three age groups: compared to the age range from our child models (see stimuli descriptions below), there was a group of younger children (6- to 9-year-olds; *n* = 33, *M* age = 8.69, *SD* = 1.03), children of the same age (10- to 11-year-olds; *n* = 24, *M* age = 10.93, *SD* = 0.50), and older children (12- to 14-year-olds; *n* = 17, *M* age = 13.03, *SD* = 0.68). Because our experiment took place at public events, we could not a priori define the number of subjects per age group. Based on previous research by van den Boomen et al. (2013), who examined visual processes in a wide age range of children, we decided that a sample size of 15 participants per age group would be sufficient to continue with the analysis.

The participants were recruited at two public events in the city of Utrecht, the Netherlands, in which the general public could learn about and participate in scientific research. The requirement to test at these events was that the complete procedure could not entail more than 15 min in total. We therefore were unable to acquire additional information about the participants, such as basic cognitive background or education level. The project was approved by the local ethics committee, and all procedures followed were in accordance with the Helsinki Declaration of 1975, revised in 2008. Participants (when 12 years or older) or their parent(s)/caregiver(s) gave informed consent at the start of the study.

### Stimuli

**Figure 1:**
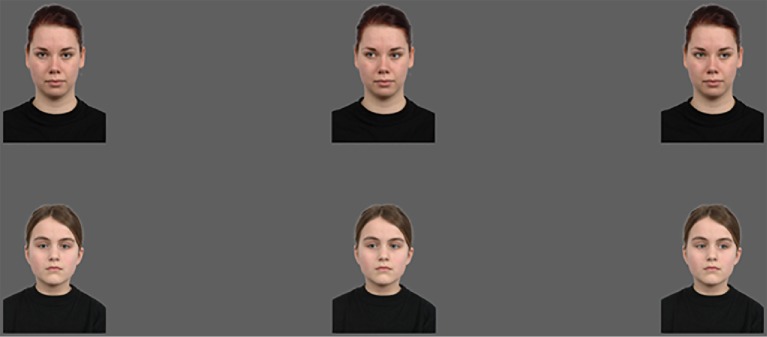
Examples of face stimuli for both adult (top row) and child (bottom row) models. Stimuli are obtained from the Radboud Faces Database (Langner et al., 2010). Written informed consent for publication was obtained.

Stimuli consisted of faces with a neutral expression taken from the Radboud Faces Database (Langner et al., 2010). We selected six adult (no. 2, 10, 19, 23, 24 and 56) and six child (no. 39, 40, 44, 63, 64 and 65) identities (each age group had three male and three female models: adult ages [21, 22, 24, 27, 29, 35]; child ages [8, 10, 10, 11, 11], one age unknown). Of each identity, we used three different pictures: with a direct gaze, left averted gaze, and right averted gaze. The pictures had a width of 600 pixels and a height of 750 pixels, with the eyes at a height of 440 pixels. Figure 1 shows examples of the stimuli used. As target pictures we used several different cartoon figures (size 300 × 300 pixels).

### Procedure

The participants visited a public scientific event and got directed to our testing booth when they expressed interest in participation in scientific research. Upon arrival, we explained our experimental set-up, without disclosing our main hypotheses, and the children, in consultation with their parents/caregivers, could decide to participate.

We conducted the gaze-cueing task with a Tobii TX300 eye tracker (sampling rate 300 Hz) integrated with a computer screen (1920 × 1080 pixels, size 24 inch; refresh rate 60 Hz). The task was programmed in Matlab version R2014b (MathWorks Inc., USA) and the Psych-Toolbox (version 3.0.12, Brainard and Vision, 1997) running on a MacBook Pro (OS X El Capitan, version 10.11.2). We positioned the participants at 65 cm distance from the screen and a chin-rest stabilized head position. After a 5-point calibration, the task started. The participants were instructed to first look at the face and then look at the target as soon as it appeared. The task consisted of 96 trials in total, 24 per condition (child/adult model; congruent/incongruent trial). First, a fixation point (50 × 50 pixels) was presented in the middle of the screen jittered between 900 and 1100 ms. Then a face with a direct gaze was presented with a random duration between 300 and 500 ms. The direct face was followed by a picture of the same face with an averted gaze to either left or right. This face (i.e., the gaze cue) was also presented for a random duration between 300 and 500 ms. Next, a target was presented at either the left or right side of the screen, which started spinning after 500 ms. It remained spinning for 1,000 ms, after which the next trial started.

### Data Reduction

We used the Identification by 2-Means Clustering algorithm (I-2MC; Hessels et al., 2017) to classify fixations. I-2MC is a fixation detection algorithm specifically designed for infant and child data with high noise levels. We interpolated periods of data loss up to 100 ms in the raw data using Steffen interpolation when at least two samples of valid data were available at each end. A moving window of 200 ms width was used for fixation classification. Fixations that were not more than 30 pixels apart and that were separated by no more than 30 ms were merged. If a fixation had a total duration shorter than 40 ms, it was removed. We excluded trials when there was no fixation on either the cue or the target (30.6% of the trials) or when the participant fixated on one side of the screen before target onset (7.3% of the trials).

If participants had less than six included trials in one or more conditions, they were excluded from analysis (*n* = 7). The final sample comprised 67 participants (34 boys; *M* age = 10.57 years, *SD* = 1.90). For each participant, the median latencies of the reaction times (RT) per condition were calculated (in ms), defined as the time between target onset and the start of the first fixation on target location.

## Results

**Figure 2:**
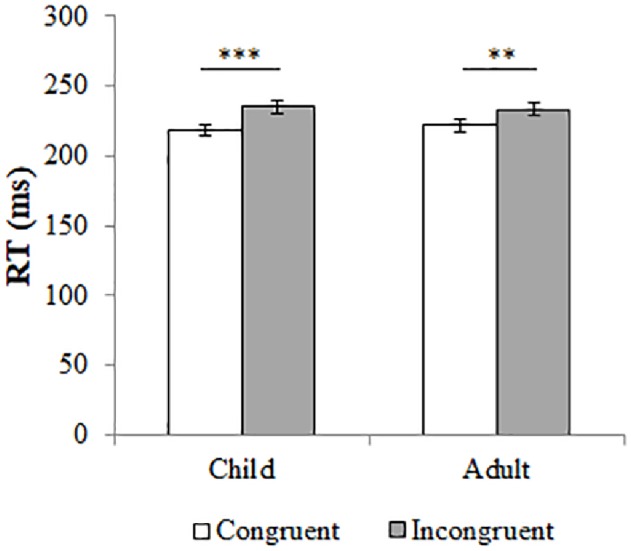
The median reaction times (RT) in milliseconds for congruent (in white) and incongruent trials (in grey). Error bars represent the standard error from the mean. Responses are separated by type of trial (either child or adult model). The difference in reaction times between congruent and incongruent trials is significant, for both child and adult models. **p < 0.005; ***p < 0.001.

We performed a repeated-measures analysis of variance (ANOVA) with congruency and age of the stimulus model as within-subjects factors.^1^ A main effect for congruency (*F*(1,66) = 16.81, *p* < 0.001, *η* ^2^ = 0.20) showed that the RTs for congruent trials (*M* = 220.33, *SD* = 35.18) were faster than those for incongruent trials (*M* = 234.21, *SD* = 35.67). There was no main effect for age of the stimulus model (*F*(1,66) = 0.09, *p* = 0.762, *η* ^2^ < 0.01) or an interaction effect between congruency and age of the stimulus model (*F*(1,66) = 1.54, *p* = 0.219, *η* ^2^ = 0.02). These results indicate that the age of the stimulus model did not influence the participants’ gaze-cueing effect. Post-hoc tests showed that for both child (congruent: *M* = 218.85, *SD* = 34.16; incongruent: *M* = 235.13, *SD* = 38.76; *t*(66) = −3.95, *p* < 0.001, Cohen’s *d* = 0.45) and adult models (congruent: *M* = 221.82, *SD* = 39.26; incongruent: *M* = 233.28, *SD* = 35.93; *t*(66) = −3.12, *p* = 0.003, Cohen’s *d* = 0.30), RTs were significantly faster for congruent compared to incongruent trials. Figure 2 shows these effects.

As the own-age effect in face recognition can appear to be very narrow (Hills and Lewis, 2011; Hills, 2012), we wanted to closely match the age of the participants with the age of the stimulus models. We therefore performed additional analyses with only a subset of the participants, that is, only the 10- and 11-year-olds (*n* = 23). Moreover, we only included those trials in which 10- and 11-year-old child models were shown (i.e., 2/3 of all child trials). A repeated-measures ANOVA showed a main effect for congruency (*F*(1,22) = 5.68, *p* = 0.026, *η* ^2^ = 0.20) with faster RTs for congruent trials (*M* = 222.47, *SD* = 41.23) compared to incongruent trials (*M* = 233.53, *SD* = 39.67). Again, we did not observe a main effect for age of the stimulus model (*F*(1,22) = 1.06, *p* = 0.314, *η* ^2^ = 0.05) or an interaction between congruency and age of the stimulus model (*F*(1,22) = 0.53, *p* = 0.528, *η* ^2^ = 0.02).

**Figure 3:**
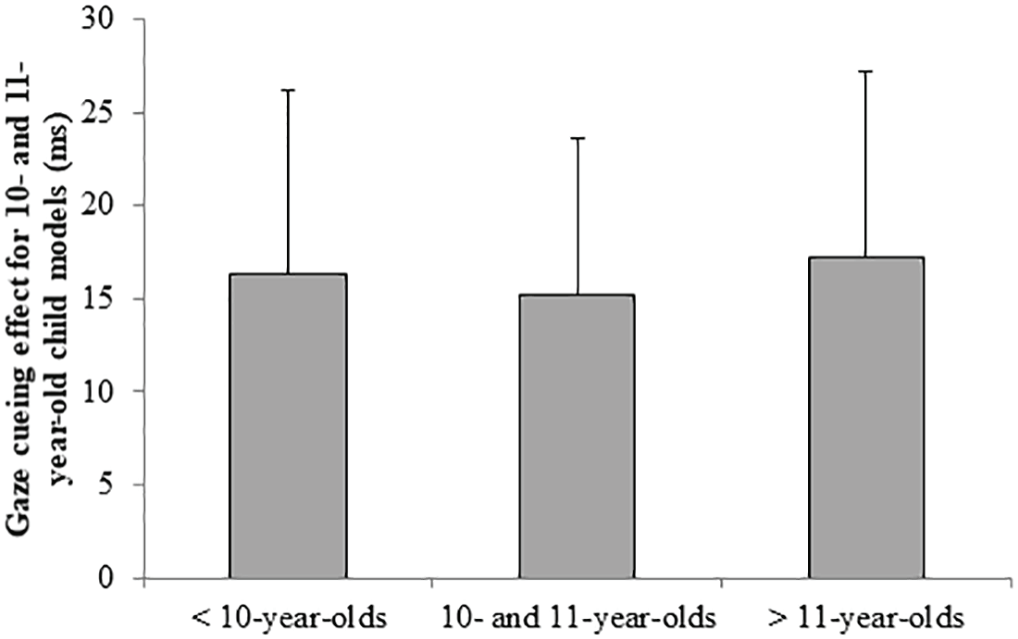
The gaze-cueing effect for only the 10- and 11-year-old child models (RTincongruent–RTcongruent) per age group. Error bars represent the standard error from the mean. There is no significant difference between the groups.

Last, we tested whether 10- and 11-year-old children differed in their gaze-cueing effect for the 10- and 11-year-old stimulus models from the younger and older participants. We performed a one-way ANOVA on the difference score for child models (RTincongruent–RTcongruent for child trials) with age group (younger [*n* = 26]; 10- and 11-year-old [*n* = 23]; older [*n* = 17]) as a fixed factor. There was no significant effect of age group on the gaze-cueing effect for child trials (*F*(2,63) = 0.01, *p* = 0.990, *η* ^2^ < 0.01). Figure 3 shows the difference scores for child models per age group. These last two analyses indicate that even for this narrow age range, there is no own-age bias in gaze cueing.

## Discussion

In the current study, we investigated whether an own-age bias is present in children performing a gaze-cueing task in which adults as well as children were our stimulus models. Evidence from literature on face recognition suggests that an own-age bias exists for visual tasks in this age group as children and adolescents show superior recognition of faces from their peers compared to adult faces (Anastasi and Rhodes, 2005; Lindholm, 2005; Crookes and McKone, 2009; Picci and Scherf, 2016). We therefore hypothesized that an own-age effect would reveal itself as stronger gaze-cueing effects for child models than for adult models. However, the present study failed to find this own-age bias in the performances on a gaze-cueing task. Even when we closely matched the age of a subsample of our participants to the age of our child models, an own-age bias was absent. Below we discuss the possibilities why the own-age bias does not appear in a gaze-cueing task testing children.

One of the explanations that might contribute to our lack of an own-age bias is the amount of exposure that participants have with seeing children and adults. The own-age bias is based on the idea that individuals have more extensive experience with individuals of their own age compared to other-aged individuals (Harrison and Hole, 2009; He et al., 2011). This is in accordance with the findings that greater familiarity with a stimulus face enhances the gaze-cueing effect (Deaner et al., 2007). Moreover, Slessor et al. (2010) demonstrated an own-age bias in gaze cueing in young adults but not in older adults. They explained this finding by suggesting that older adults may have greater experience with people of different age ranges, whereas younger adults have more contact with people of their own age. Future studies should entail measures of experience with different age groups to assess the specific effect of experience on the own-age bias in gaze cueing.

Children might be in a similar situation as older adults, as they too, are surrounded by people of different age ranges. Young children are from birth focused on attachment with their primary caregiver. They are also around same-aged peers in day care and later in school, yet adult faces remain important in daily life. Much of children’s learning occurs in the context of social interactions with adults (i.e., parents, teachers, and trainers), which involves joint attention processes. The coordination of one’s own perspective and another person’s perspective (based on that person’s eye gaze) relative to a third object or event provides experiences that are fundamental to social-cognitive neurodevelopment (Mundy and Newell, 2007; Mundy and Jarrold, 2010; Mundy, 2016). Only in puberty, adolescents reorient specifically from adults toward peers (Scherf and Scott, 2012). With a mean age of 10.4 years, our sample of children has possibly not reached this point of reorientation yet. In other words, while it is possible that they increasingly focus more on other children, they would still highly focus on adults as well. It is possible that this balance could contribute to the lack of an own-age bias in the present study. This would entail that social interactions with either adults or peers are equally important in learning situations during late childhood, with similar effects on social-cognitive neurodevelopment. Longitudinal designs could shed light on the moment in development where the own-age bias in gaze cueing arises, if it does at all. This would give more information about the moment in development where interactions with peers are valued as more important than interactions with adults and thus possibly also the moment in development where interactions with peers have a larger influence on social-cognitive neurodevelopment.

Another potential explanation for the observed lack of an own-age bias in the current study is related to differences in the underlying cognitive processes of gaze cueing and face recognition. While it is in the face recognition literature that own-age biases are frequently reported, gaze cueing is a notably different process than face recognition. Evidence from studies investigating event related potentials (ERPs) highlight that both processes have different time courses. The own-age bias in face recognition is expressed in the N250, a peak around 250 ms after image presentation which reflects activation of facial representations for recognition (for a review, see Wiese et al., 2013). In contrast, gaze direction is processed slightly earlier, that is, between 150 and 200 ms after gaze motion onset (Conty et al., 2007; Itier and Batty, 2009). It is therefore possible that the neural processes underlying the own-age bias operate too slow to affect gaze cueing. Yet such an explanation conflicts with other studies that testify the presence of an own-age bias in gaze-cueing tasks, albeit with young and older adults (Slessor et al., 2010; Bailey et al., 2014). Clearly, at least for adults, there appears to be an interaction between mechanisms underlying gaze cueing and age processing. It is possible that adults’ perceptual processes have been matured enough to rapidly integrate multiple sources of information that a model possibly conveys. In other words, while adults might simultaneously integrate and evaluate a model’s age with the direction of the eyes, children might only become aware of a model’s age after they have processed the model’s eyes. In our sample of children, perceptual processes are still expected to be developing (e.g. van den Boomen et al., 2012), and therefore, the effect of a model’s age might not yet reach these rapid perceptual processes.

The current study has some limitations as well. First, we conducted a post-hoc power analysis to determine the reliability of our results. We have to note that the observed power for the interaction between congruency and age of the stimulus model is 0.23. In the analysis with only the 10- and 11-year-old participants, the observed power for this interaction is 0.11. These values are rather low, and therefore, replication is key. At the moment, we cannot conclude with high certainty that there is no own-age bias in gaze cueing in children. Future studies, possibly with higher sample sizes, should replicate the current study to come to more reliable conclusions. Second, we only recorded eye-tracking data and obtained no other behavioral response times such as key presses. This makes our results comparable with infant gaze-cueing studies, where it is common to calculate reaction times based on eye gaze data. However, our results are less comparable to adult gaze-cueing data, as these data are mainly acquired through key presses.

We still looked at former studies to get an indication of the magnitude of gaze-cueing effects over different ages. In our own study with children, we observed a gaze-cueing effect of 16.28 ms [95% CI (8.06, 24.51)] for child models and an effect of 11.46 ms [95% CI (4.13, 18.79)] for adult models. The young adult participants in the study of Slessor et al. (2010) show a gaze-cueing effect of 19.95 ms for young adult models and an effect of 12.00 ms for old adult models, whereas the older participants show gaze-cueing effects of 8.73 and 13.19 ms, respectively. Last, when only assessing the results with supraliminal neutral stimuli in the study of Bailey et al. (2014), we observe a gaze-cueing effect of 14.1 ms for young adult models and an effect of 6.3 ms for old adult models in young adult participants, whereas the older participants showed effects of 4.2 and 5.4 ms, respectively. We notice here that, although not always significantly, all age groups show the largest gaze-cueing effects for stimuli of their own age. When comparing gaze-cueing effects for young adult stimulus models, we notice that young adult participants display the largest gaze-cueing effect. The children in our own study show a slightly smaller effect than the young adults in the other two studies, whereas the older adult participants show notably smaller gaze-cueing effects for these young adult stimulus models than the other two age groups. This observation is in accordance with the theory that more extensive experience with individuals of a certain age results in a bias for this category of faces (Harrison and Hole, 2009; He et al., 2011). Young adults are the ones who at this stage of their lives have most experience in viewing other young adults. However, when comparing the results of these three studies, we have to keep the procedural differences between these studies in mind. Whereas we used eye-tracking data, the other two studies used key presses to calculate reaction times. It is possible that this difference in responses makes the reaction times, and therefore the gaze-cueing effects, less comparable. Moreover, there are other differences in experimental set-up. First, we used a picture of a face with direct gaze before the onset of the averted gaze, which was not used in the other two studies. Second, the time between the onset of the averted gaze cue and the onset of the target differed between studies. These differences might influence reaction times as well.

Overall, the results of the present study highlight that children’s responses to gaze cues are not modulated by the age of the stimulus model, similar to findings in senior adults (Slessor et al., 2010). This might indicate that children consider models of either adults or of their own age as equally informative, and therefore, they put the same amount of effort in processing these gaze cues. In contrast, children might also be insensitive to a model’s age and disregard age information in the gaze-cueing process. A gaze cue on itself might have enough relevance to be processed effectively, regardless of the person one is looking at. Future research should aim at trying to disentangle these two possibilities and shed light on the possible processes taking place when reacting to gaze cues. Yet, the current results highlight that, at least in late childhood, age of the stimulus model does not modulate gaze-cueing effects.

## Data Availability Statement

The raw data supporting the conclusions of this manuscript will be made available by the authors, without undue reservation, to any researcher.

## Ethics Statement

This study was carried out in accordance with the recommendations of ethical guidelines of the local Ethics Committee of the Faculty of Social Sciences, Utrecht University. All subjects (when 12 years or older) or their parent(s)/caregiver(s) gave written informed consent in accordance with the Declaration of Helsinki. The protocol was approved by the Ethics Committee of the Faculty of Social Sciences, Utrecht University.

## Author Contributions

RR and CJ contributed to the conception and design of the study; RR was responsible for the data collection, data processing, and data analyses; and RR wrote the first draft of the manuscript. All authors contributed to the manuscript revision, read, and approved the submitted version.

### Conflict of Interest Statement

The authors declare that the research was conducted in the absence of any commercial or financial relationships that could be construed as a potential conflict of interest.

## Footnotes

^1^We also performed the repeated-measures ANOVA with age as a covariate. However, age did not have a linear effect on any variable or a main effect on RTs. We therefore decided to report the simpler analysis without age as a covariate.
